# Direct Temperature Responses of *Bmal1* and *Per2* in Reindeer Fibroblasts: Insight Into a Unique Circadian Clock Adaptation to Polar Environments

**DOI:** 10.1177/07487304251327871

**Published:** 2025-03-24

**Authors:** S. K. Tahajjul Taufique

**Affiliations:** Department of Neuroscience, UT Southwestern Medical Center, Dallas, Texas

A sense of time is crucial for the survival of organisms. Most organisms have evolved with circadian clocks to entrain their behavior and physiology with the daily rhythms of their environment. These internal timekeepers entrain to rhythmic external cues, particularly the reliable 24-h light-dark cycle. However, in polar regions, continuous daylight prevails during summer and continuous darkness dominates during winter. Under these extreme lighting conditions, some animals (e.g. porcupines and ground squirrels; Folk et al., 2006) continue to show nearly 24-h rhythmic activity. In contrast, other animals (e.g. reindeer and Svalbard rock ptarmigans; Stokkan et al., 1986, van Oort et al., 2005) have arrhythmic behavior when light cycles are absent, but become rhythmic during spring and autumn when light-dark cycles are present. The mechanisms that underlie the seasonal switches between arrhythmic and rhythmic behaviors are unknown. In 2010, Lu et al. investigated whether the molecular circadian timekeeping oscillatory mechanism was intact in reindeer that had arrhythmic activity in constant lighting conditions. They measured circadian gene expression from cultured reindeer skin fibroblasts using luciferase reporters and observed low amplitude and rapidly damping bioluminescence rhythms. These findings suggested that reindeer lack a functional molecular clock which could explain the arrhythmic activity during summer and winter. However, it was uncertain whether their rhythmic activity during spring and fall is a direct response to the light-dark cycle or is governed by the circadian clock.

A recent study from Appenroth et al. (2024) adds new insights into the molecular circadian clock in reindeer. In contrast to the initial study of reindeer molecular rhythms, Appenroth et al. showed that reindeer skin fibroblasts have a functional molecular clock. They found that reindeer fibroblasts exhibit temperature-compensated circadian rhythms in the promoter activity of both *Bmal1::luc* and *Per2::luc*. In constant culture conditions, *Bmal1::luc* and *Per2::luc* rhythms had the typical antiphase relationship. Interestingly, Appenroth et al. uncovered a unique response of reindeer fibroblasts to temperature cycles (37.2 °C/40.2 °C). Under these conditions, fibroblasts exhibited a rapid and atypical in-phase expression pattern for *Bmal1::luc* and *Per2::luc* rhythms. This atypical phase relationship in reindeer fibroblasts was verified by measuring *Bmal1* and *Per2* mRNA. The atypical phase response to temperature cycles may be specific to reindeer since mouse fibroblasts exhibited the typical anti-phasic expression of *Bmal1* and *Per2* in the same conditions.

The researchers further explored this phenomenon by mutating the retinoic acid-related orphan receptors response element (RORE) in the mouse *Bmal1* promoter. *Bmal1-mutated::luc* was arrhythmic under constant temperature conditions, but, surprisingly, had an atypical temperature-driven rhythm in the temperature cycle. This atypical expression profile was not limited to reindeer cells as mouse and human (U2OS) cells with mutated RORE also had atypical temperature-driven rhythms. These results suggest the presence of 2 distinct regulatory mechanisms driving *Bmal1* transcription: (a) circadian clock-dependent rhythmic transcription through RORE and (b) direct temperature regulation through an unknown motif, which enables *Bmal1* to exhibit temperature-driven rhythmicity. These findings suggest that rhythmic activity in reindeer during spring and fall is regulated by the circadian clock that entrains to the light-dark cycle. Perhaps during summer and winter, when light-dark information is absent, circadian gene expression responds directly to environmental temperature which causes the atypical phases of *Bmal1* and *Per2* rhythms. This atypical in-phase relationship could suppress the circadian clock in reindeer and that might lead to arrhythmic behavior.

The results further imply that circadian clock-dependent regulation of Bmal1 transcription is weaker in reindeer compared to mice. In reindeer fibroblasts, temperature-driven, atypical *Bmal1* expression rhythms dominate under the temperature cycle. In mouse fibroblasts and U2OS cells, this atypical *Bmal1* expression rhythm is revealed only after circadian clock-dependent regulation is disabled by mutating the RORE in the *Bmal1* promoter. These findings raise the question: what mechanism regulates temperature-driven *Bmal1* transcription? One possible mechanism could be that heat shock factors (HSFs) directly bind to unknown regulatory regions in the *Bmal1* promoter and regulate temperature-driven transcriptions, as has been shown for the *Per2* promoter (Tamaru et al., 2011). Novel temperature-sensitive motifs could be identified by investigating the effects of truncating different parts of the *Bmal1* promoter on temperature-driven rhythms in reindeer fibroblasts.

Species-specific differences in promoter sequences or transcription factors and cofactors may account for the differences in the atypical temperature response of *Bmal1* between reindeer and mice. This could be investigated by using a reindeer-specific *Bmal1* promoter to transduce both reindeer and mouse cells. If the reindeer-specific promoter exhibits an atypical response to temperature cycles in both reindeer and mouse cells, then it could indicate a difference in the promoter sequence between the species. On the other hand, if the atypical temperature response is seen only in reindeer cells, then species-specific transcription factors or cofactors might be responsible. Comparative analysis of the promoter sequence and factors or co-factors could provide insights into the novel temperature-driven mechanism.

Finally, the differences in findings between Appenroth et al. and Lu et al. are unexplained at present. The culture conditions and synchronization procedure were similar in both studies. In addition, the reporter constructs in both studies had the same key regulatory elements and included insulator sequences that shielded from positioning effects. A possible difference between the studies that might have influenced the circadian rhythm is the number of passages of the fibroblast cells. Appenroth et al. used P6-8, while Lu et al. did not report the passage numbers. Another possible difference is the timing of the experiments. Appenroth et al. collected the tissues on February 27 (the start of spring), whereas Lu et al. may have conducted their experiment during Fall, but the date is not reported. It is possible there are seasonal changes in fibroblasts that affect the molecular clock to make it rhythmic or arrhythmic. Epigenetic modifications, such as chromatin accessibility, DNA methylation, or histone modifications, could mediate these seasonal changes. This seasonal shift hypothesis could be tested by comparing molecular rhythms in reindeer fibroblasts harvested at different times of the year. Investigation into the molecular clock in other polar animals could provide deeper insights into the circadian clock adaptation to the polar environment.
